# In-patient psychiatry management of COVID-19: rates of asymptomatic infection and on-unit transmission

**DOI:** 10.1192/bjo.2020.86

**Published:** 2020-09-01

**Authors:** Emily Zhang, Elizabeth LeQuesne, Katherine Fichtel, David Ginsberg, W. Gordon Frankle

**Affiliations:** NYU Grossman School of Medicine, New York, New York, USA; Department of Psychiatry, NYU Grossman School of Medicine, and NYU Langone Health, New York, New York, USA; Department of Psychiatry, NYU Grossman School of Medicine, and NYU Langone Health, New York, New York, USA; Department of Psychiatry, NYU Grossman School of Medicine, and NYU Langone Health, New York, New York, USA; Department of Psychiatry, NYU Grossman School of Medicine, and NYU Langone Health, New York, New York, USA

**Keywords:** In-patient treatment, phenomenology, risk assessment, disease transmission, COVID-19

## Abstract

**Background:**

New York City's first case of SARS-associated coronavirus (SARS-CoV-2) disease 2019 (COVID-19) was identified on 1 March 2020, prompting rapid restructuring of hospital-based services to accommodate the increasing numbers of medical admissions. Non-essential services were eliminated but in-patient treatment of psychiatric illnesses was necessarily maintained.

**Aims:**

To detail the response of the NYU Langone Health in-patient psychiatric services to the COVID-19 outbreak from 1 March to 1 May 2020.

**Method:**

Process improvement/quality improvement study.

**Results:**

Over this time period, our two in-patient psychiatric units (57 total beds) treated 238 patients, including COVID-19-positive and -negative individuals. Testing for COVID-19 was initially limited to symptomatic patients but expanded over the 62-day time frame. In total, 122 SARS-CoV-2 polymerase chain reaction (PCR) tests were performed in 98 patients. We observed an overall rate of COVID-19 infection of 15.6% in the patients who were tested, with an asymptomatic positive rate of 13.7%. Although phased roll-out of testing impaired the ability to fully track on-unit transmission of COVID-19, 3% of cases were clearly identified as results of on-unit transmission.

**Conclusions:**

Our experience indicates that, with appropriate precautions, patients in need of in-patient psychiatric admission who have COVID-19 can be safely managed. We provide suggested guidelines for COVID-19 management on in-patient psychiatric units which incorporate our own experiences as well as published recommendations.

After identifying its first case of SARS-associated coronavirus (SARS-CoV-2) disease 2019 (COVID-19) on 1 March 2020,^1^ New York City (NYC) rapidly became the epicentre of the pandemic in the USA, with 172 354 positive cases as of 1 May 2020.^2^ Over the course of 2 months, hospitals made rapid changes to accommodate the increasing number of patients requiring admission and intensive care unit (ICU) support for COVID-19 by expanding the number of beds available and eliminating non-urgent procedures and admissions.^3,4^ Despite the combination of increasing numbers of patients with COVID-19 requiring admission and the reduction or elimination of non-urgent hospital services, patients with psychiatric illness continue to require in-patient admission for management and stabilisation of severe symptoms, including psychosis, depression and suicidality. In fact, the stressors of the pandemic increase the risk of exacerbation of patients’ underlying psychiatric conditions, particularly for those unable to attend their regular out-patient appointments or access critical medications, including long-acting injectable antipsychotic medications.^5^

The high infectivity of SARS-CoV-2 carries many challenges to maximising safety in hospitals, but the standard treatment provided on in-patient psychiatric units poses many additional and unique difficulties in the management of transmissible diseases. A core part of the milieu treatment on in-patient psychiatric units involves interacting in close proximity with other patients and staff. Traditionally, patients are encouraged to spend time out of their rooms, taking part in group therapy, meals in common dining spaces, art and recreational groups, and interactions with multidisciplinary treatment teams. While removing these activities decreases viral transmission within the unit, it simultaneously removes an important component of patients’ in-patient stabilisation. Preventing infectious spread is even more of a challenge in patients who have difficulty following isolation or personal protective equipment (PPE) protocols, either owing to loss of reality testing and inability to understand the gravity of the situation, or because of difficulties with self-control.^6^ Evidence of these challenges can be seen in reports of COVID-19 outbreaks on in-patient psychiatric units in Wuhan, China,^7^ Cheongdo, South Korea,^8^ and Seattle, Washington, USA.^9^

In the context of the COVID-19 outbreaks on in-patient psychiatric units, several authors have proposed guidelines for changes in care delivery aimed at reducing on-unit transmission and management of COVID-19 among psychiatry in-patients.^6,10–13^ We report the experience at NYU Langone Health in implementing these measures with the goal of balancing the, at times, opposing forces to provide quality care for patients’ mental health while protecting their physical health.

## Overview of NYU Langone Health in-patient psychiatry

The in-patient psychiatric services at NYU Langone Health are made up of two units totalling 57 in-patient beds. The first, HCC-10, is a 22-bed voluntary unit, located in Tisch Hospital in midtown Manhattan. The second, LB5900, is a 35-bed voluntary/involuntary unit, located in NYU Langone Hospital Brooklyn, in the Sunset Park neighbourhood of Brooklyn. Combined admissions for these two units in 2019 were 1687 (730 to HCC-10; 957 to LB5900), with average lengths of stay of 9.3 days on HCC-10 and 11.3 days on LB5900. HCC-10 serves a predominantly English-speaking (96.9%) patient population with either private, managed care insurance, or the government-funded programmes Medicaid (a federal-state assistance programme for low-income Americans) or Medicare (a federal insurance programme for elderly and disabled Americans); 35% of the unit's patients are covered by Medicaid/Medicare. The unit also takes care of patients with medically complex problems and a high proportion of patients referred from New York University's (NYU's) undergraduate Counseling and Wellness Services at the NYU Student Health Center. In contrast, as a part of a community-based hospital in Brooklyn, LB5900 serves a population of patients who are insured predominantly through the government-funded programmes Medicaid/Medicare (69%), with a larger proportion (17.1%) of patients whose primary language is not English.

This paper gives the results of an ongoing process improvement/quality improvement study underway at NYU Langone Health.

## Method

### Instituting preventive measures

The in-patient psychiatric services of NYU Langone Health started to change their approach to in-patient treatment in response to the COVID-19 pandemic in the first week of March 2020. The changes made, outlined below, are largely consistent with recently published guidelines/recommendations^6,10–13^ and remained in place throughout the period described.

- Initially, the number of visitors was reduced to one per patient, with visitors screened for temperature and infectious symptoms in the hospital lobby before entering the unit, per hospital protocol. Within the next week, no visitors were allowed on the in-patient psychiatry units, to reduce transmission.
- Hand sanitiser dispensers were mounted on the walls to provide small metred doses to patients.
- ‘High-touch’ surfaces in common areas (such as door handles and telephones) were cleaned hourly.
- Group therapies were initially attempted with social distancing; however, this proved impractical and face-to-face group therapy was therefore eliminated after 2 weeks.
- Meals were delivered to patients’ rooms, rather than being served in a common dining area.
- Medications were distributed room-to-room rather than at a central location.
- Access to common areas was reduced and patients were encouraged to spend time in their rooms, minimising contact with peers.
- Patients were asked to wear hospital-provided surgical masks when in the common areas.
- The process for holding mental health court hearings for involuntary commitment and medications over objection (compulsory treatment) was transitioned to video hearings.
- The electroconvulsive therapy (ECT) service for HCC-10 was temporarily closed when the infection rate and medical hospital admissions were at their highest, in an effort to reduce transmission as well as to accommodate critical care demands for space and personnel. The service was reopened as demand for ECT increased, and patients who received ECT during this period were tested and had to be COVID-19-negative by polymerase chain reaction (PCR).

To mitigate the adverse impact of these measures, patients on LB5900 were provided with individual electronic devices (Android tablets) with newspapers, streaming entertainment services (Netflix, Hulu, Live TV), games and music. On HCC-10, where unit protocol allows for patients to use personal electronic devices as clinically appropriate, patients also received single-use art therapy kits to use in their rooms. Patients employed technology at both sites to video conference with family and friends, as well as to have video visits with members of the clinical team who were working remotely. On HCC-10, the staff covered/taped up the cameras on patients’ personal electronic devices to maintain privacy on the unit. On LB5900, the video functionality of the devices was restricted to select video conferencing software controlled by the hospital's information technology group with video conferences between patients and family/friends established by staff. On both HCC-10 and LB5900, individual therapy, physician meetings and one-to-one meetings between patients and other staff continued, with staff using appropriate PPE and maintaining social distancing. All staff wore surgical masks or N95 respirators on the unit and when interacting with COVID-19-negative patients. While interacting with COVID-19-positive patients, staff wore gowns, gloves, N95 respirators and eye protection (‘contact, droplet and eye precautions’). The PPE was removed when moving from a COVID-19-positive patient to a COVID-19-negative patient using standardised donning/doffing techniques (see supplementary material available at https://doi.org/10.1192/bjo.2020.86).

The staff workflow was also restructured to minimise potential transmission between staff members and patients and among staff members themselves. With the dissolution of group therapy, licensed creative art therapists were pulled from the unit, followed by the administrative staff. In mid-March, all staff meetings were transitioned from face-to-face to video conference; at the same time, NYU medical students were removed from their clinical rotations on HCC-10. In early April, a portion of the in-patient attending physicians began to work remotely via video meetings, by either continuing to see patients on the psychiatric unit (LB5900) or transitioning to the hospital's consultation liaison service (HCC-10), which had observed an increased volume of consultations. Resident physicians were redistributed similarly to participate in patient care via telepsychiatry. By mid-April, a portion of the in-patient psychiatry social work team also began to work remotely.

Census management (bed management) was a critical aspect to allow for social distancing on the in-patient units. In March, patients who were psychiatrically stabilised were discharged as early as possible, provided that appropriate out-patient supports could be put into place. The census on both units decreased and remained low owing to the decreased number of people leaving their homes and presenting to the emergency department. Additionally, with universities transitioning to online classes, there was no longer the regular influx of college students referred from the NYU Student Health Clinic to HCC-10. This provided the opportunity for each patient on HCC-10 to have their own room, while on LB5900 it allowed for a four-room (eight-bed) section of the unit to be maintained for COVID-19-positive patients. Both units continued to admit patients regardless of their COVID-19 status as long as the patients were medically stable and without supplemental oxygen requirements; patients requiring supplemental oxygen were admitted to the medical services. Over the course of the peak 2 months of COVID-19 in NYC (from 1 March to 1 May 2020), 238 patients had a total of 253 admissions to the in-patient psychiatric units at NYU Langone Health, a reduction of 22% from the same time period in 2019, with decreases of 35% for HCC-10 and 9% for LB5900. In addition, there were 44 patients who were admitted prior to 1 March 2020 and received in-patient care on the units for some portion of time after this date. We did not note any difference in payer mix during this period. The legal statuses at the time of admission, the patient demographics and diagnoses are provided in Table 1.

**Table 1:** Characteristics for patients admitted to the two NYU Langone Health in-patient psychiatric units (HCC-10 and LB5900) between 1 March and 1 May 2020

	HCC-10	LB5900	Total
Patients, *n*	93	145	238
Voluntary admission status, %	100.0	28.9	55.3
Age, years: mean (s.d.)	37.9 (17.2)	41.6 (15.9)	40.1 (16.5)
Female, %	60.2	46.2	51.7
Ethnicity, %			
White	51.6	31.0	39.1
African American	8.6	16.6	13.4
Asian/Pacific Islander	7.5	10.3	9.2
Other/unspecified	32.3	40.7	37.4
Undomiciled, %	9.6	26.4	20.2
Diagnoses, %			
Suicidal ideation/attempt	37.6	8.3	19.7
Depression	37.6	17.2	25.2
Schizophrenia/psychosis	57.9	79.0	54.3
Bipolar disorder	5.4	13.1	10.1
Other diagnoses	42.9	37.6	23.2
COVID-19 positive, %^a^	4.3	9.0	7.1

a. Represents the proportion of known COVID-19-positive patients treated during this period (*n* = 13 on LB5900; *n* = 4 on HCC-10). Before 6 April 2020, not all patients received a COVID-19 test.

### Patient monitoring and testing for SARS-CoV-2

The monitoring and testing of patients evolved over the course of 1 March to 1 May 2020. Initially, owing to low testing resources, tests for SARS-CoV-2, the virus that causes COVID-19, by PCR were only administered to patients with current or recent symptoms, including fever, cough and shortness of breath. Screening for these symptoms occurred both in the emergency department and on the in-patient units. On the in-patient units, increased medical surveillance of patients for these symptoms began in early March and included increased monitoring of vital signs: three times a day on LB5900 and twice a day on HCC-10. In the absence of strict guidelines for when to utilise COVID-19 PCR testing, patients with non-specific symptoms such as fatigue and low-grade fevers (below 38°C) were tested on a case-by-case basis when there was clinical suspicion for COVID-19. Patients were also tested if their roommate tested positive, regardless of symptoms. By early April 2020, on the basis of recommendations by infectious disease physicians at NYU Langone Health, any patient who had a temperature of 37.2°C or higher was tested. These new criteria expanded the number of patients who received COVID-19 testing; however, all tests in this category were negative. The increased number of patients who had received tests, in combination with the rapidly increasing availability of tests within NYU Langone Health, prompted a change in the guidelines to conduct surveillance testing of all patients admitted to in-patient psychiatry as of 6 April 2020. Patients on the units who had not already been tested for COVID-19 were tested at that time. In response to the increased recognition of asymptomatic carriers detected via universal screening, all new admissions were isolated and placed under ‘contact, droplet and eye precautions’ while screening tests were pending. Often, COVID-19 screening tests were able to be performed in the emergency department prior to arrival on the in-patient psychiatric units. In the cases where the test results were pending or the tests were not completed at the time the patient arrived on the in-patient unit, patients were isolated in their own rooms on HCC-10 or in a room allocated for patients with pending tests on LB5900.

After the initiation of COVID-19 screening tests for all patients, repeat testing based on symptoms was implemented if a patient who initially tested negative developed a temperature over 37.8°C or other potential symptoms of COVID-19, including cough and shortness of breath. Patients were immediately isolated in their own rooms on HCC-10 or in the room allocated for patients with pending tests on LB5900 until their status was clarified. If the patient under investigation shared a room with another patient, their roommate was also retested, regardless of clinical symptomatology. In the rare case that a patient refused testing, they were isolated as a patient under investigation until a test could be obtained.

### Management of COVID-19-positive patients

Patients who tested positive for COVID-19 were placed under contact, droplet and eye precautions and asked to isolate themselves in their rooms on the unit. On HCC-10, patients remained in their own individual rooms, whereas on LB5900, the in-patient unit was restructured so that a block of four rooms (eight beds) at the end of one hallway was allocated for COVID-19-positive patients. COVID-19-positive patients were cohorted by gender if necessary and clinically appropriate. Very few problems arose with patients being unable to comply with isolation protocols; when this did occur, the patient was redirected by staff to their room and, at times, place on on-to-one supervision until able to follow safety measures. As clinically appropriate, medications were administered as needed in response to behavioural control problems. If at any time a patient on either unit developed serious symptoms requiring oxygen support, they were transferred to the medicine department for continued management, with consultation liaison psychiatry monitoring their psychiatric status. Removal of patients from isolation and contact/droplet/eye precautions with integration into the non-COVID portion of the unit(s) was implemented following steps outlined in the Appendix (point (3)d).

### Ethical approval

The data contained in this paper were collected as part of ongoing process improvement/quality improvement and did not require NYU Langone Health institutional review board review.

## Results

### COVID-19 testing of patients

In the 62 days from 1 March to 1 May 2020, 98 patients received 122 tests for COVID-19 on the in-patient psychiatric units of NYU Langone Health. Of those, 81 patients had a single test on the unit and 17 patients had more than one test. The repeat tests were performed for a variety of reasons, including new onset of symptoms (*n* = 6 tests, 2 positive), discontinuing isolation (*n* = 6 tests, 4 positive), temperature between 37.2–37.7°C during the time when testing was indicated for patients with temperatures in this range (*n* = 6 tests, 0 positive), exposure to a COVID-19-positive patient (*n* = 3 tests, 0 positive) and confirmation of a negative test (*n* = 3 tests, 0 positive). In addition, 2 patients, both on HCC-10, were accepted for admission with known COVID-19-positive status and were not retested on admission and therefore not included in the data-set.

**Fig. 1:**
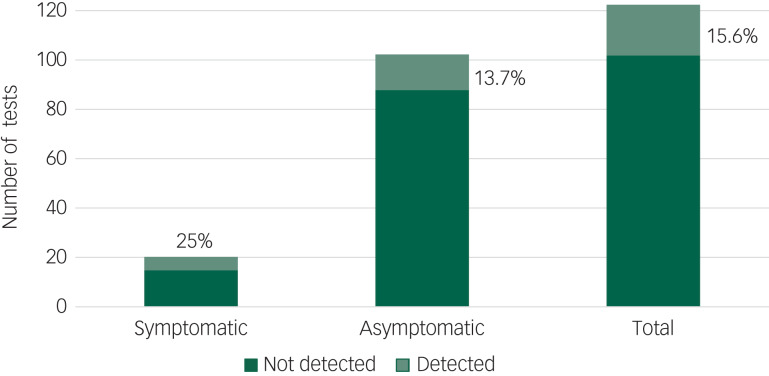
All COVID-19 tests of patients in in-patient psychiatric services at NYU Langone Health, New York City.

Figure 1 shows the results of all tests performed on the NYU Langone Health in-patient psychiatry units during this period; overall, COVID-19 was detected in 15.6% of the tests performed overall, and 13.7% of the tests performed on asymptomatic patients.

All COVID-19 polymerase chain reaction (PCR) tests performed between 1 March and 1 May 2020 on the two NYU Langone Health in-patient psychiatric units (HCC-10 and LB5900). Negative tests are shown in dark green and positive tests are shown in light green, with the percentage of positive tests denoted on the graph. Overall, we observed a positive test rate of 15.6% (19 of 122).

**Fig. 2:**
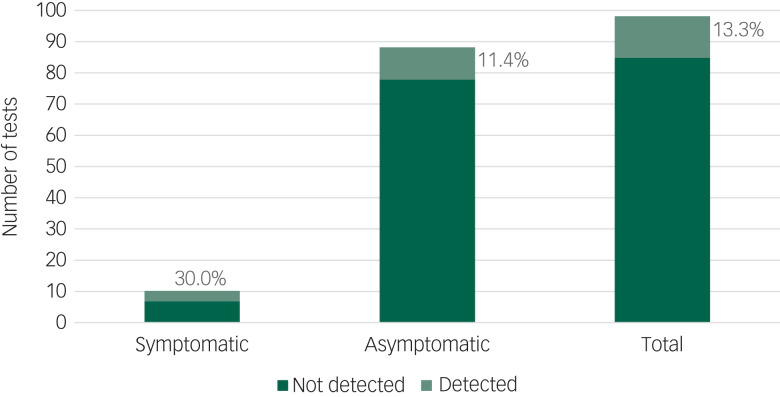
Initial COVID-19 tests of patients in in-patient psychiatric services at NYU Langone Health, New York City.

Figure 2 shows the results of only the initial COVID-19 tests for each patient; in this case we observed a 13.3% overall positive test rate, with 11.4% of the tests resulting positive in asymptomatic patients. In total, 88 patients received an initial COVID-19 test while asymptomatic, with 3 of these tests being done in the context of direct exposure to a COVID-19-positive roommate.

Initial COVID-19 polymerase chain reaction (PCR) tests for each patient performed between 1 March and 1 May 2020 on the two NYU Langone Health in-patient psychiatric units (HCC-10 and LB5900). Negative tests are shown in dark green and positive tests are shown in light green, with the percentage of positive tests denoted on the graph. Overall, we observed a positive test rate of 13.3% (13 of 98).

### On-unit transmission

Identifying clear cases of on-unit transmission in the time frame between the 1 March 2020 and the implementation of surveillance/unit-wide testing on 6 April 2020 is difficult, given an incubation period for COVID-19 of up to 14 days^14,15^ and the existence of asymptomatic infections/carriers.^16,17^ To examine on-unit transmission before the implementation of surveillance testing, we identified all patients who had their first COVID-19 test after having already been admitted to the in-patient unit. Thirty-three patients had a first test that fell into this category, with an average length of time between admission and the COVID-19 test of 13.9 days (s.d. = 15.7). Twenty-five out of these 33 tests were negative (75.8%), and the average time between admission and testing was 14.1 days (s.d. = 17.7, range 2–61 days). The 8 patients with positive tests had an average time between admission and testing of 13.1 days (s.d. = 7.2; the times for the respective patients were 5, 7, 9, 11, 14, 15, 16 and 28 days) and may represent cases of on-unit transmission. However, 5 of these 8 patients were asymptomatic at the time of the test. Additionally, the absence of a negative test at the time of admission, in combination with reports showing that SARS-CoV-2 PCR tests may remain persistently positive for over 1 month,^18^ makes it impossible to state definitively whether these 5 cases represent on-unit transmission or not. The 3 patients who were symptomatic at the time of the test had been on the in-patient unit for 5, 11 and 28 days respectively. Since the median incubation period of COVID-19 is 5.1 days,^14^ with 97.5% of patients developing symptoms in 11.5 days, at a minimum, it can safely be assumed that the patient who had been on the unit for 28 days represents a case of on-unit transmission.

The implementation of surveillance testing on 6 April 2020 allowed for much clearer identification of on-unit transmission. Two patients on LB5900, in unrelated events, tested positive having previously tested negative during their admission – both developed low-grade fevers, which prompted retesting. The first was a 43-year-old female admitted with suspected Korsakoff syndrome or autoimmune encephalitis, who was exhibiting ongoing disorganised behaviour on the unit and was difficult to redirect. On day 75 of her admission, she developed a temperature of 38.1°C, prompting retesting, which was positive; her previous negative COVID-19 test was 7 days earlier. Since her previously negative test status converted to positive after 75 days as an in-patient, it is likely that she was exposed to SARS-CoV-2 from patients or staff. The second was a 65-year-old female admitted with bipolar affective disorder, manic state with psychosis, who also exhibited disorganised behaviour on the unit. On day 25 of her admission, she developed a temperature of 38.1°C, prompting retesting, which was positive; her previous negative COVID-19 test was 21 days earlier. Both patients remained on the in-patient psychiatric unit throughout their admission.

### Rates of staff infection

A total of 133 individual staff worked on the in-patient psychiatry units of NYU Langone Health in the period from 1 March to 1 May 2020. Of these individuals, 24 (18.0%) developed known COVID-19 infections. The criteria for testing of staff evolved over this time. Initially, staff members were only tested if they developed symptoms; subsequently, testing was expanded to include testing in the case of exposure to a positive individual. Given this, it is likely that some staff had asymptomatic infection. Table 2 provides details of the infection rates for the different disciplines.

**Table 2:** COVID-19 infection rates for psychiatric staff on the two NYU Langone Health in-patient psychiatric units (HCC-10 and LB5900) between 1 March and 1 May 2020

	% testing positive (staff total, *n*)		
	HCC-10	LB5900	Combined
All staff	14.6 (45)	20.0 (85)	18.0 (133)
Registered nurses	17.2 (29)	19.0 (42)	18.3 (71)
Patient care technicians	12.5 (8)	19.2 (26)	17.6 (34)
Social workers	33.3 (3)	50.0 (6)	44.4 (9)
Administrative staff	0 (2)	25.0 (4)	16.7 (6)
Licensed creative arts therapists	0 (3)	0 (3)	0 (6)
Physicians	0 (3)	0 (4)	0 (4)

## Discussion

Management of any transmissible disease can be challenging on in-patient psychiatric units, given the mobility of the patient population and the therapeutic use of common spaces for dining and gathering, peer-to-peer interactions and group treatments. COVID-19 has specific challenges, owing to the relatively long asymptomatic period and the increasing recognition that individuals with few or no symptoms can still spread the illness.^14,17^ Over the course of the peak 2 months of the COVID-19 crisis in NYC (1 March to 1 May 2020), NYU Langone Health managed a total of 238 in-patients on its psychiatry units. We were able to progressively implement steps to reduce on-unit spread of the virus. These changes were largely successful, with the caveat that at least two patients developed COVID-19 while on the in-patient unit after the implementation of surveillance testing. In addition, there was one the patient who did not have an initial negative test but, given the time between admission and the development of COVID-19 symptoms (28 days), was highly likely a case of on-unit transmission. Taken together, this provides a minimum rate of on-unit transmission of 3% (3 out of 98). Finally, we describe other potential cases of on-unit transmission that were more ambiguous owing to the lack of prior negative tests or consistent surveillance testing during the admission processes at the time these cases were observed. This underscores the critical role of surveillance testing in the management of COVID-19 on in-patient psychiatric units: without knowing a patient's baseline COVID-19 status when admitted, it is unclear whether infections were acquired before or after their admission to in-patient psychiatry, since these patients who tested positive later on might have experienced a longer than usual incubation period (for those who developed symptoms) or might be asymptomatic carriers. Of note, since patients were not routinely tested on discharge, there may have been other patients who developed an asymptomatic infection on the units that went undetected.

Comparing the two units, we saw overall higher rates of COVID-19-positive tests on LB5900 (18.5%) than on HCC-10 (6.7%), probably due to the high number of involuntary admissions on LB5900, since involuntary status tends to be an indicator of more severe mental illness with greater average length of stay. In two of the three cases of on-unit transmission occurring on LB5900, the patient's clinical state was characterised by a high degree of disorganisation, creating difficulties for them in complying with staff instructions or with safety measures on the unit (e.g. social distancing, hand hygiene, wearing masks). It is interesting that we did not clearly observe the opposite: a patient with a high degree of disorganisation spreading the illness throughout the unit, nor did we observe a spike in infections after each of these patients was noted to have COVID-19. In other words, their level of disorganisation appeared to contribute more to their contracting the virus than to spreading the virus. Although this remains speculative, it is consistent with the notion that an individual's behaviours play a significant role in reducing the risk of contracting COVID-19.^19^ In line with this, the vast majority of patients were able to follow the safety measures put in place and experienced the changes to the unit as largely positive in response to the crisis. This was true even in the case of individual patients who were symptomatic with serious mental illnesses, including schizophrenia.

### Comparison with infection rates on a NYC obstetrics unit

One interesting aspect of this report is the rate of asymptomatic COVID-19-positive tests. We observed that 13.7% of all SARS-CoV-2 PCR tests performed in asymptomatic individuals were positive (Fig. 1). This rate is identical to a recent report in which Sutton et al noted a 13.7% positive test rate of asymptomatic patients screened on admission for delivery at an obstetrics unit in NYC.^20^ A limitation of this comparison is that some of the tests performed in asymptomatic patients on our units were done in response to proximity to known COVID-19-positive patients, something that is less likely to occur on an obstetrics unit. When we examined only the initial tests, we found that 11.4% of asymptomatic patients’ initial tests were positive. The discrepancy between our rate of 11.4% and the rate from Sutton et al may be explained by the lack of universal screening for all of March on our units, potentially causing other asymptomatic COVID-19-positive patients to remain undetected. Additionally, obstetric patients are usually admitted for a few days, whereas psychiatric patients are admitted for a longer period, allowing for an increased observation period during which symptoms may develop. The data published by Sutton et al were collected over a period (22 March to 4 April 2020) that overlapped with that of the current study. The similarity of these two rates in two very different patient populations, collected at the same time in the same city, lends credence to our conclusion that, despite the unique challenges of infection prevention on in-patient psychiatric units, the rate of on-unit transmission can be maintained at a low rate with proper precautions and universal testing. The similarities also support the conclusion that the prevalence of carriers of SARS-CoV-2 could be in the range of 14% of the asymptomatic population in areas where widespread community transmission exists.^21^

### Staff infection rates

Finally, we included data on the rates of staff infections for informational purposes and to provide context to the data derived from patients. Owing to the limited sample size, the infection rate among all staff overall is more reliable than the rates within each discipline. It should be noted that the rates of staff infections were higher than those seen in patients on our in-patient units and not statistically significantly different across the units (unpaired *t*-test, statistical data not shown). Furthermore, the staff infection rate is likely to have been even higher than that stated because initially only symptomatic employees were tested. This is not unexpected since only patients with minimal or no COVID-19 symptoms were admitted to the psychiatry units, whereas those with medically significant symptoms were admitted to the medical services. In addition, once admitted to the units, patients were in a controlled environment with a high degree of safeguards and mitigation strategies in place to reduce transmission on the unit, whereas, in their time outside the unit, staff were exposed to all the factors that contributed to NYC becoming a hot spot of the COVID-19 outbreak. The differing rates of infection in patients and staff raises the question as to whether or not transmission occurred between patients and staff, in either direction. Unfortunately, it is not possible to determine whether this did in fact take place, a point highlighting the need for frequent, universal testing of both patients and hospital staff in the context of widespread community transmission. This is particularly true in settings such as in-patient psychiatric units with mobile patient populations, where contact, eye and droplet precautions cannot be taken with each patient encounter.

### Management implications

As experts such as Center for Disease Control and Prevention Director Robert Redfield have stated, we are anticipating subsequent waves of SARS-CoV-2 infection over the next years until a vaccine or effective treatment is readily available.^22,23^ Therefore, it will be even more crucial to deploy early effective means to prevent the spread of COVID-19 on in-patient units in the future to accommodate for increased patient influx as state-wide and local social distancing and quarantining policies are relaxed. The NYU Langone Health experience indicates that, with appropriate precautions, patients in need of in-patient psychiatric admission who have COVID-19 can be safely managed. The Appendix provides suggested guidelines for COVID-19 management on in-patient psychiatric units which incorporate our own experiences as well as those previously published.^6,10–13^

## Data Availability

The data that support the findings of this study are available from the corresponding author, W.G.F., on reasonable request.

## Supplementary material

For supplementary material accompanying this paper visit https://doi.org/10.1192/bjo.2020.86. click here to view supplementary material

## Appendix

Suggested guidelines for in-patient psychiatry management of COVID-19

1. Routine testing for all admissions
  - Patients should be under droplet/eye/contact precautions and in designated room(s) while awaiting test results.
2. Monitoring development of COVID-19 symptoms on the unit
  - Increase the frequency of temperature checks to 2–3 times a day.
  - Retest patients with temperature >37.8°C or who develop other symptoms of COVID-19 (e.g. cough, dyspnoea, anosmia, ageusia).^24^
  - Place patients under droplet/eye/contact precautions while awaiting test results.
  - If the patient is not in a single room, move the patient to a designated room while awaiting test results, if possible.
  - When appropriate, retest the roommate of patients who test positive.
3. Managing patients who test positive for COVID-19
  - Maintain COVID-19-positive patients in a designated area of the unit.
  - Place COVID-19-positive patients under droplet/eye/contact precautions.
  - Limit the number of staff physically interacting with COVID-19-positive patients.
  - Remove patients from precautions/isolation who meet all the following criteria:^25^
    - passage of at least 3 days (72 h) from resolution of fever without the use of antipyretics
    - improvement in respiratory symptoms (e.g. cough, shortness of breath)
    - passage of at least 7 days from symptom onset
    - negative results of at least two consecutive COVID-19 tests collected at least 24 h apart
    - for patients who were asymptomatic at the time of their first positive test and remain asymptomatic, testing for release from isolation may begin a minimum of 7 days from the first positive test.
4. Modified procedures for patient care
  - Eliminate in-person visitors.
  - Encourage social distancing and hand hygiene for patients.
  - Encourage patients to wear surgical masks when outside of their rooms.
  - Eliminate or minimise the use of shared spaces, including elimination of meals in a common dining area.
  - Use technology for video visits with physicians.
  - When possible, increase the use of video conferencing for one-to-one therapy and group therapy.
  - Increase entertainment options.
5. Modified staffing and operations
  - Routinely monitor and test staff.
  - Reduce on-unit staffing whenever possible for disciplines that can operate remotely.
  - Enforce that staff wear masks at all times.
  - Increase cleaning of unit, focusing on ‘high-touch’ areas, including telephones, doors, tablets, etc.
  - Hold mental health court hearings for involuntary commitment and treatment over objection (compulsory treatment) via video.
  - Stock and replenish five sets of personal protective equipment (PPE) on the unit for behavioural emergencies with COVID-19-positive patients.
  - Consider temporary suspension of the electroconvulsive therapy (ECT) service, depending on infection rates and larger hospital demands, balancing these with the psychiatric patient care needs.
