# Supplementary material for: In-patient psychiatry management of COVID-19: rates of asymptomatic infection and on-unit transmission
Source: BJPsych Open. 2020 Sep 1;6(5):e99. doi: 10.1192/bjo.2020.86 (PMC7463133; doi:10.1192/bjo.2020.86)
Supplement: Supplementary file 1 [file S2056472420000861sup.zip › S2056472420000861sup001.pdf]

Checklist for doffing PPE

Step to check off	Activity	Location of activity	Detail	
1 ☐	Remove gown and gloves first<br><br>Roll gown into itself, peeling off gloves at same time.<br>Hold gown away from your body and discard in regular waste	Do this in doorway inside the room or in anteroom if available	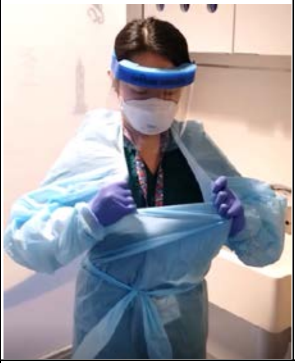	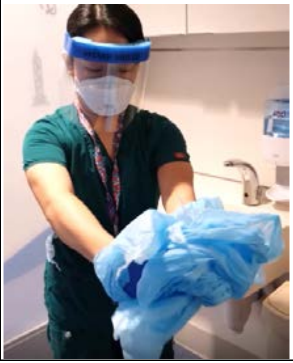
2 ☐	Perform Hand hygiene		Use hand sanitizer	
3 ☐	Remove and discard face shield or goggles. Avoid touching front of face shield or goggles<br><br>Discard in regular waste	Do this in doorway inside the room or in anteroom if available	Remove face shield by grasping sides of strap and then pull forward over head<br>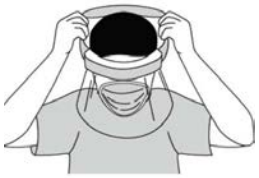	Remove goggles by grasping sides and pull away from your face<br>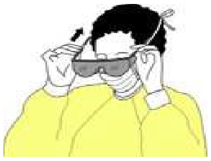
4 ☐	Perform Hand hygiene		Use hand sanitizer	
5 ☐	Remove and discard N-95 respiratory. Avoid touching front of N95<br><br>Discard in regular waste	Outside of patient room or anteroom	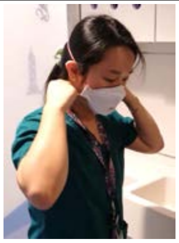	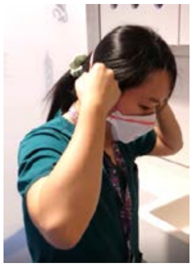
6 ☐	Perform Hand hygiene	Outside of room	Perform hand hygiene with alcohol-based hand rub or soap and water	
